# Identification and Functional Characterization of the GPAT Gene Family in Regulating Oil Biosynthesis in Olive

**DOI:** 10.3390/plants15152295

**Published:** 2026-07-27

**Authors:** Shengjia Huang, Lihua Wang, Min Ye, Wanbo Wu, Hui Luo, Lingfan Yu, Pijun Li, Yang Huang, Jincheng Du

**Affiliations:** Sichuan Academy of Forestry, Chengdu 610081, China; huangshengjia0206@163.com (S.H.); wwlhbb@163.com (L.W.); yemincome@163.com (M.Y.); 66880027@163.com (W.W.); paul779375110@163.com (H.L.); yulingfan328@163.com (L.Y.); barophiles@139.com (P.L.); huangyang0927@163.com (Y.H.)

**Keywords:** olive, GPAT, oil synthesis

## Abstract

Olive (*Olea europaea* L.) is one of the oldest woody oil crops. Previous studies, by integrating transcriptomics and metabolomics, have identified glycerol-3-phosphate acyltransferase 4 (*GPAT4*) as a key regulatory gene involved in oil biosynthesis in olive fruits. Although *GPAT4* was identified as a key regulator, the overall function of the *GPAT* gene family in olive remains largely unknown. As a critical rate-limiting enzyme catalyzing triacylglycerol production, *GPAT* plays an important role in oil synthesis in oil crops. However, the function of the *GPAT* gene in olive remains unclear. A systematic analysis of the olive *GPAT* gene family showed that 12 OeGPAT proteins can be divided into four subclasses, all containing the conserved *GPAT* domain. These genes are distributed across chromosomes and scaffolds, with highly conserved motifs and variable gene structures. Functional studies revealed that overexpression of *OeGPAT1.1*, *OeGPAT1.2*, *OeGPAT2*, *OeGPAT3*, *OeGPAT4*, *OeGPAT7*, and *OeGPAT8* significantly increased total oil content in transgenic *Arabidopsis* leaves and seeds. Further experiments in olive fruit at maturity stages II, III, and IV demonstrated that overexpression of the same set of *OeGPAT* genes elevated total oil content, while their silencing resulted in a decrease. In summary, this study reveals that *OeGPATs* can serve as ideal targets in metabolic engineering to regulate oil content in oil crops, providing a theoretical foundation for further research on oil biosynthesis in olive fruit.

## 1. Introduction

Olive (*Olea europaea* L.), an evergreen tree of the family Oleaceae and genus Olea, is a diploid species with a chromosome number of 2n = 2x = 46, and is recognized as one of the world’s four major woody edible oil crops [1]. Following more than 60 years of introduction and cultivation, olive has become a well-established industry in China. According to the *China Olive Industry Development Blue Book (2024)*, the national olive planting area expanded from 1.35 million mu (approximately 90,300 ha) in 2018 to 2.18 million mu (approximately 145,000 ha) by the end of 2024, representing a growth of about 60.6% over six years. By 2024, fresh olive fruit production reached 108,000 tons, and olive oil output exceeded 12,000 tons, both showing significant year-on-year increases. The market share of domestically produced olive oil has risen significantly, indicating a gradual reduction in import dependence, and China is steadily emerging as a new olive-oil-producing country. Nevertheless, compared with leading olive-producing countries worldwide, China’s olive industry still faces considerable gaps in both scale and economic efficiency. Therefore, increasing olive oil yield and enhancing industrial profitability remain crucial objectives for the high-quality development of China’s olive industry [2].

Olive oil, extracted from olive fruits, is rich in bioactive components such as unsaturated fatty acids, vitamins, and polyphenols, conferring high nutritional, medicinal, and economic value [3]. The monounsaturated oleic acid is the most abundant fatty acid in olive oil, accounting for 56.0~79.0% of total lipids. The other fatty acids which are highly represented in the olive oil composition are: palmitic (10.10~19.28%) and linoleic (2.68~22.15%) [4]. Olive oil can help prevent certain chronic diseases and effectively delay aging [5]. The presence of various antioxidant components, especially polyphenolic flavonoids, is one of the key reasons why olive oil is regarded as a “functional oil” [6]. These compounds play a vital role in protecting the human body from oxidative damage caused by free radicals.

The synthesis and metabolism of oils in olive are exceptionally complex, involving intricate regulatory relationships among multiple metabolic pathways during fruit growth, development, and ripening. Olive fruit development exhibits a distinctive double-sigmoid curve pattern, with the entire developmental process lasting approximately five months [7]. During this period, oil synthesis only gradually initiates once the olive fruit reaches a certain developmental stage [8]. Within 30–40 days after pollination, olive fruits undergo rapid growth primarily due to cell proliferation and begin to accumulate substantial amounts of carbohydrates, providing both energy and substrates to support subsequent oil synthesis. Around 80 days after pollination, the fruit enters another phase of rapid growth driven by cell expansion. As the fruit matures and enlarges, its oil content progressively increases, peaking at full maturity [9]. Recent research indicates that oil biosynthesis in olive fruit is closely linked to the maturation process, a phenomenon also observed in other plant species [10]. This developmental phase is marked by significant biochemical changes, involving the synthesis and interconversion of various metabolites that critically influence final oil yield [11].

Given the urgent need to improve both the yield and quality of olive oil in China, elucidating the molecular mechanisms governing oil biosynthesis in olive fruit is of great importance. A key enzyme in this process is glycerol-3-phosphate acyltransferase (GPAT), which catalyzes the acylation of glycerol-3-phosphate (G3P) at the *sn*-1 position to form lysophosphatidic acid (LPA). This initial reaction constitutes the committed and rate-limiting step in the triacylglycerol (TAG) biosynthetic pathway [11]. In plants, oils are primarily synthesized and stored as TAGs in seeds and fruits. TAGs not only support plant growth and development but also provide energy for seed germination [12]. The biosynthesis of TAG begins with an acylation reaction catalyzed by GPATs; therefore, GPAT is a key rate-limiting enzyme in the TAG synthesis pathway, and its activity directly influences the composition and accumulation of oils [13]. The *GPAT* gene family is widely present in eukaryotes, and all its members contain a conserved phosphate acyltransferase domain. This family not only participates directly in oil biosynthesis but also plays a central role in the positive regulation of plant responses to abiotic stress, such as drought and salinity [14,15]. Increasing evidence suggests that transcription factors serve as central regulatory nodes that integrate multiple stress and developmental pathways to enhance plant resilience [16].

To date, varying numbers of *GPAT* genes have been identified in different plant species, such as *Arabidopsis* (10 members) [17], maize (20 members) [18], rice (18 members) [19], and soybean (28 members) [20]. In *Arabidopsis*, the 10 *GPAT* members are classified into three groups based on their subcellular localization: The first group comprises mitochondrial *GPATs*, including *AtGPAT1* to *AtGPAT3*. Among them, *AtGPAT1* possesses sn-2 acyltransferase activity and is essential for pollen development [21]. The second group consists of endoplasmic reticulum (ER) *GPATs*, including *AtGPAT4* to *AtGPAT9*. *AtGPAT4* and *AtGPAT8* are key enzymes for cutin biosynthesis [22]. *AtGPAT5* is crucial for suberin synthesis in roots and seed coats [23]. *AtGPAT6* is involved in cuticle formation in floral organs and affects stamen development [24]. *AtGPAT7* may play a role in wound-induced suberin synthesis [25]. *AtGPAT9* directly participates in TAG biosynthesis and is positively correlated with seed oil content [26]. The third group contains plastidial *GPATs* represented by *AtATS1*, which is mainly involved in the biosynthesis of phospholipids, sulfolipids, and galactolipids [27]. The essential role of *GPATs* is well-documented: for instance, in peanut, overexpression of *AhGPAT9* enhances seed oil content, while its knockdown reduces it, and allelic variations in this gene are significantly associated with oil content [27]. Moreover, *PfGPAT9* has been shown to be crucial for the biosynthesis of the valuable, polyunsaturated fatty acid-rich oil in Perilla frutescens seeds [28].

In contrast to the extensive studies in other oil crops, the *OeGPAT* gene family in olive (*Olea europaea* L.) has not been systematically characterized, and the functional roles of its members in oil biosynthesis remain largely unknown. Our previous integrated transcriptomic and metabolomic analyses (BioProject accession number CRA038923) identified *OeGPAT4* as a key regulator of oil biosynthesis in olive fruits, suggesting that other *OeGPAT* members may also play important roles. Based on these findings, the present study aimed to: (i) systematically identify the *OeGPAT* gene family in the olive genome and analyze their phylogenetic relationships, gene structures, and expression patterns; (ii) functionally characterize the key *OeGPAT* members involved in oil biosynthesis using heterologous expression in model plants and transient transformation in olive fruits; and (iii) evaluate their potential as targets for metabolic engineering to improve oil yield and quality. The results of this study will fill a critical knowledge gap in the molecular regulation of olive oil biosynthesis and provide candidate genes for the genetic improvement of olive and other woody oil crops.

## 2. Results

### 2.1. OeGPAT4: A Key Gene Regulating Oil Metabolism and Oil Quality in Olive

In a previous study (unpublished), comprehensive widely targeted metabolomics and Illumina RNA-seq analyses were performed on five olive cultivars at different maturity stages to investigate the relationship between oil synthesis and the expression of related genes. The results showed that the total oil accumulation in the ‘Arbequina’ cultivar at maturity stage III was significantly higher than that in other cultivars and at other maturity stages.

Integrated transcriptomic and lipidomic analyses revealed that *OeGPAT4* was differentially expressed between the two comparison groups and closely associated with olive oil metabolism, particularly glycerophospholipid synthesis. The expression pattern of this gene was stage-specific: it was up-regulated at maturity stage III compared with stage II, but down-regulated at stage IV relative to stage III, suggesting its potential role in mediating oil metabolic differences across maturation phases. *OeGPAT4* regulates phosphatidylcholine (PC) synthesis, thereby influencing both membrane lipid composition and the supply of lipid precursors. It provides unsaturated acyl groups for triglyceride assembly, positioning it as a key node linking membrane lipid metabolism and storage oil biosynthesis. The differential expression of *OeGPAT4* may represent an important molecular mechanism underlying the differences in oil quality between maturity stage III and the other stages. Thus, *OeGPAT4* emerges as a candidate gene for modulating oil metabolism or for varietal improvement in olive. Accordingly, we further conducted a comprehensive analysis of its gene family.

### 2.2. Analysis of the Olive GPAT Gene Family

**Figure 1:**
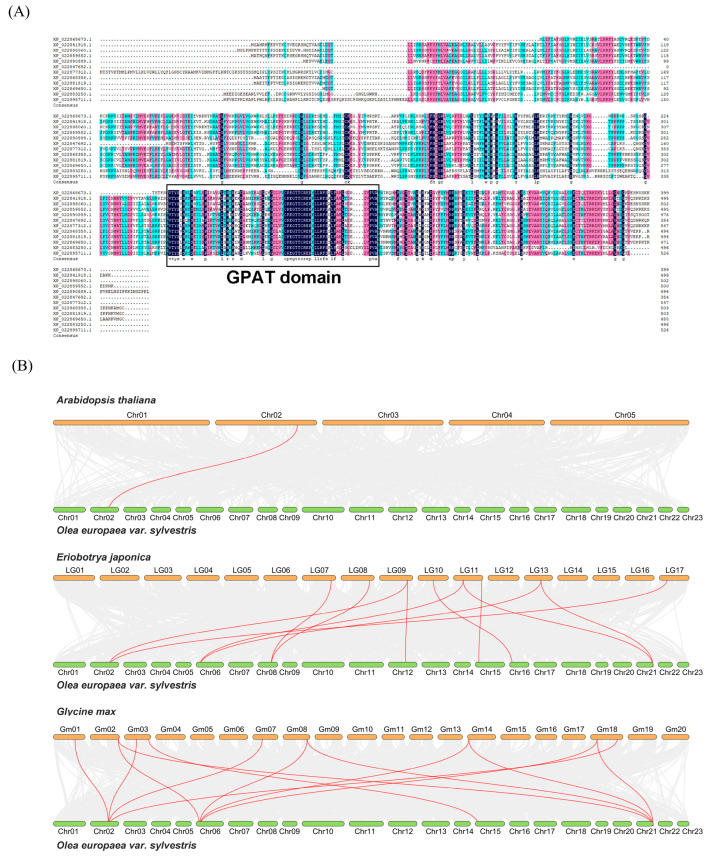
Comparative analysis of conserved domains and cross-species homology of olive GPAT proteins. (**A**) Sequence alignment of 12 OeGPAT proteins from olive. Shaded areas indicate highly conserved amino acid residues such as glycine (G), serine (S), and proline (P). The OeGPAT domain (amino acids 247–288) is highlighted by a black box. (**B**) Synteny analysis of *OeGPAT* gene families among olive, *Arabidopsis*, loquat, and soybean. Gray lines indicate genomic syntenic blocks; red lines connect orthologous gene pairs.

On the basis of the above findings, we further performed a genome-wide identification and systematic analysis of the olive *GPAT* gene family. Multiple sequence alignment of the 12 OeGPAT amino acid sequences obtained from the whole genome was performed using DNAMAN 10.3 software. The results showed that all 12 OeGPAT proteins contain a GPAT domain, which occupies most of the protein sequence (positions 247–288 aa) and includes several highly conserved amino acid residues such as Glycine (G), Serine (S), and Proline (P) (Figure 1A). Evolutionary homology analysis of the *OeGPAT* gene family among olive, *Arabidopsis*, loquat, and soybean was performed using MCScanX. Gray areas indicate syntenic regions between olive and the other species across different chromosomes, while red areas highlight syntenic relationships specifically involving *OeGPAT* gene family members. The analysis identified 1 orthologous gene pair between olive and *Arabidopsis*, 11 orthologous pairs between olive and loquat, and 13 orthologous pairs between olive and soybean (Figure 1B).

**Figure 2:**
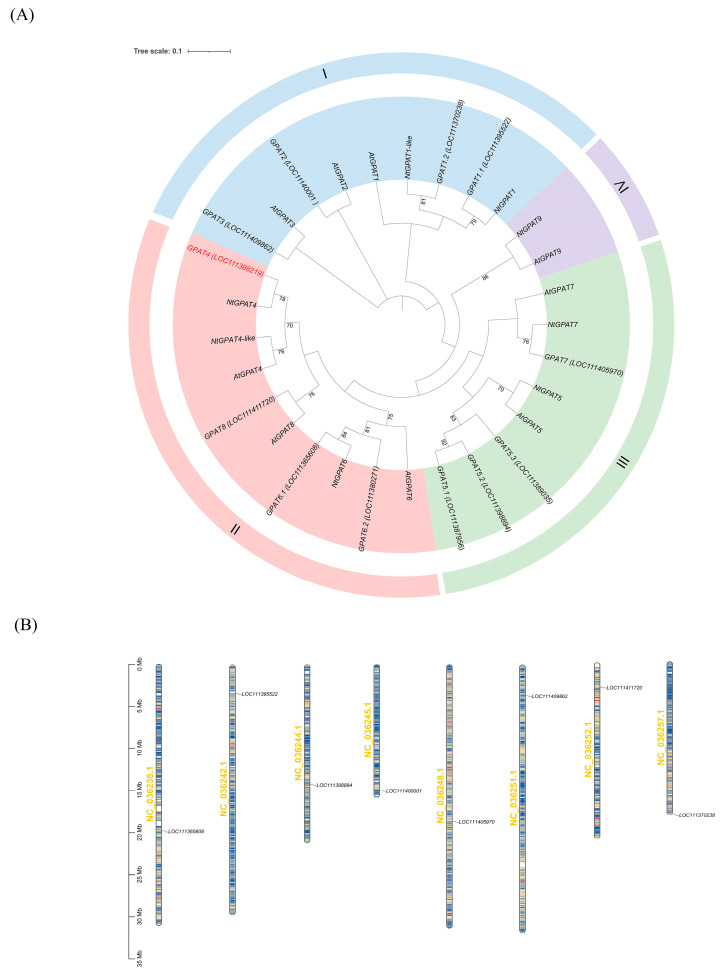
Phylogenetic analysis and chromosomal localization of olive *GPAT* genes. (**A**) Phylogenetic tree of GPAT proteins from *Arabidopsis* and olive constructed using the maximum likelihood method. Olive *GPAT* members are classified into four subfamilies (I–IV). The target gene *OeGPAT4* (LOC111388219) belongs to Group I. (**B**) Chromosomal distribution of olive *GPAT* genes. Eight genes are located on eight chromosomes, while the remaining four (including *OeGPAT4*, LOC111388219) reside on unanchored scaffolds.

A phylogenetic tree was constructed using Maximum Likelihood (ML) method in MEGA 12.1, trimAl v1.5.1, and IQ-tree 3.1.3 software based on aligned GPAT protein sequences from *Arabidopsis* and olive. According to the established subfamily classification of *Arabidopsis GPAT* genes, the 12 olive GPAT proteins were classified into four clades: Class I contained 4 OeGPAT proteins, Class II contained 4 members, Class III contained 2 members, and Class IV contained 2 members. LOC111388219 was identified as *OeGPAT4*, which catalyzes the initial step of glycerooil synthesis and is a key enzyme in the triglyceride biosynthesis pathway (Figure 2A). Chromosomal localization of the olive *GPAT* gene family was visualized using TBtools. Based on genome annotation file (GFF3) analysis, 8 *OeGPAT* genes were distributed across 8 chromosomes, while the remaining 4 *OeGPAT* genes, including *OeGPAT4* (LOC111388219), were located on unanchored scaffolds (Figure 2B).

**Figure 3:**
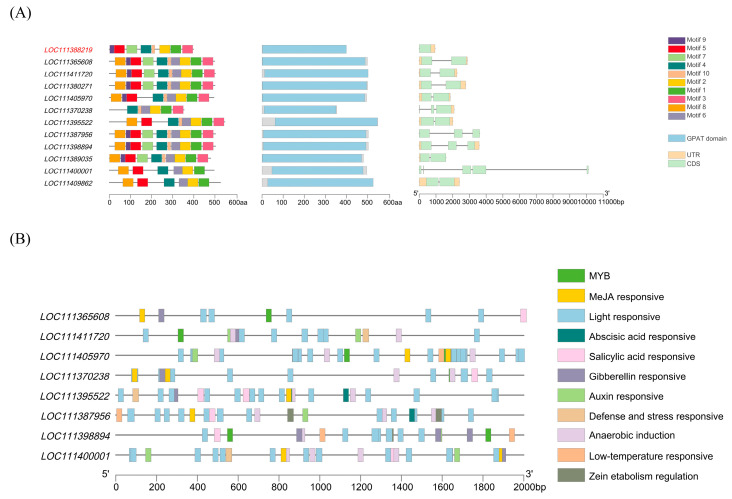
Structural features and cis-acting element analysis of the olive *GPAT* gene family. (**A**) Analysis of conserved motifs, domains, and gene structures of the *OeGPAT* gene family. Boxes of different colors represent distinct motifs; gray boxes indicate domains; yellow boxes and black lines represent exons and introns, respectively. (**B**) Analysis of cis-acting elements in the promoter regions of the eight *OeGPAT* genes located on chromosomes. Shapes of different colors represent various types of cis-acting elements, such as light-responsive, hormone-responsive, and stress-responsive elements.

Conserved motif analysis revealed that the 12 *OeGPAT* genes are highly conserved in most motifs. Most *OeGPAT* genes contain motif1, motif2, motif3, and motif4. The absence of certain motifs in some *OeGPAT* genes may be due to partial loss during olive genome evolution. LOC111388219 contains the four motifs present in most *OeGPAT* genes but lacks motif6 and motif8, suggesting it may have distinct biological functions and may have evolved independently, possibly through genetic events such as gene duplication that led to changes in motif composition. Domain analysis showed that the *OeGPAT* domain distribution is relatively consistent across most genes, occupying the majority of the protein sequence. Gene structure analysis indicated that the number of exons in *OeGPAT* genes ranges from 1 to 5, and the number of introns ranges from 0 to 4. LOC111400001 contains an exceptionally large intron that may play an important role in gene structure, potentially influencing gene expression by increasing splicing diversity or harboring regulatory elements. Variations in the number and distribution of introns and exons among members suggest differentiation within the *OeGPAT* gene family (Figure 3A). Cis-acting element analysis of the 8 *OeGPAT* genes located on chromosomes identified four main types of elements in addition to basic elements (CAAT-box and TATA-box): hormone response elements, light response elements, stress response elements, and physiological response elements. A significant number of light response elements were found in the 8 *OeGPAT* genes, followed by anaerobic induction elements, suggesting that these genes are involved in light response regulation and are also influenced by anaerobic stress (Figure 3B).

### 2.3. Functional Modulation of Selected GPATs Alters Petroleum Ether Extract Weight in Tobacco

**Figure 4:**
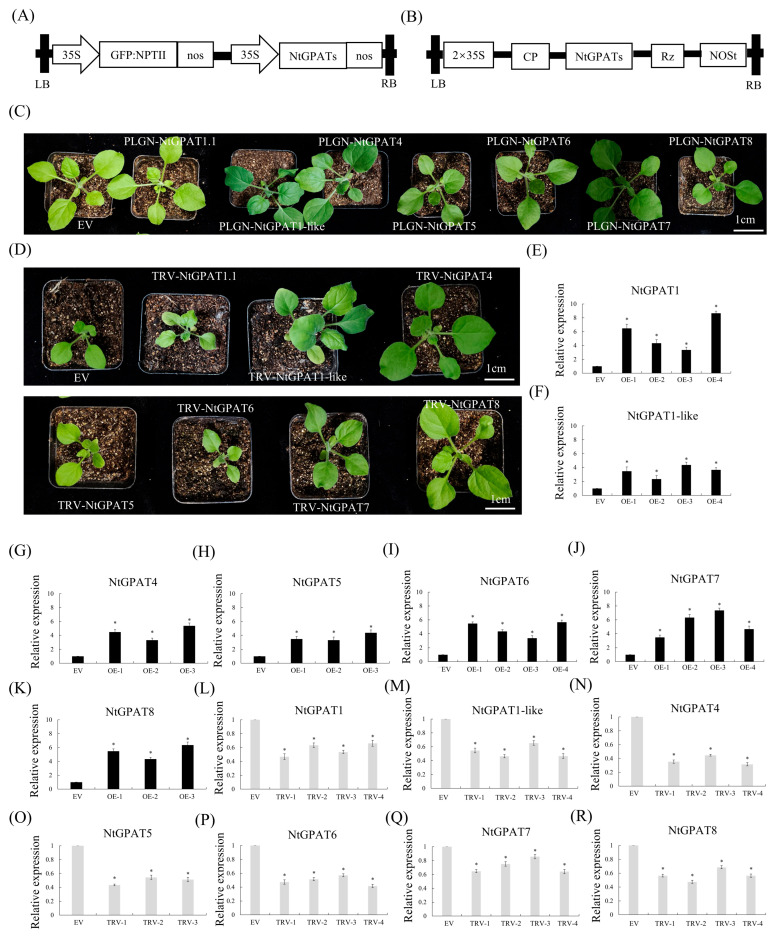
Construction and expression validation of *NtGPAT* overexpression and silencing systems in tobacco. (**A**) Construction of the overexpression vector. (**B**) Construction of the TRV vector. (**C**) *NtGPAT*-overexpressing tobacco plants. (**D**) *NtGPAT*-silenced tobacco plants (transient). Scale bar: 1 cm. (**E**–**K**) Relative gene expression levels in *NtGPAT*-overexpressing lines. EV: empty vector tobacco leaves (control). (**L**–**R**) Relative gene expression levels in transiently *NtGPAT*-silenced plants. Scale bar: 1 cm. Error bars indicate ± SD with three replications. A single asterisk (*) specifies that the difference is significant between the treatment and the control at *p* < 0.05 (Duncan’s multiple range test).

**Figure 5:**
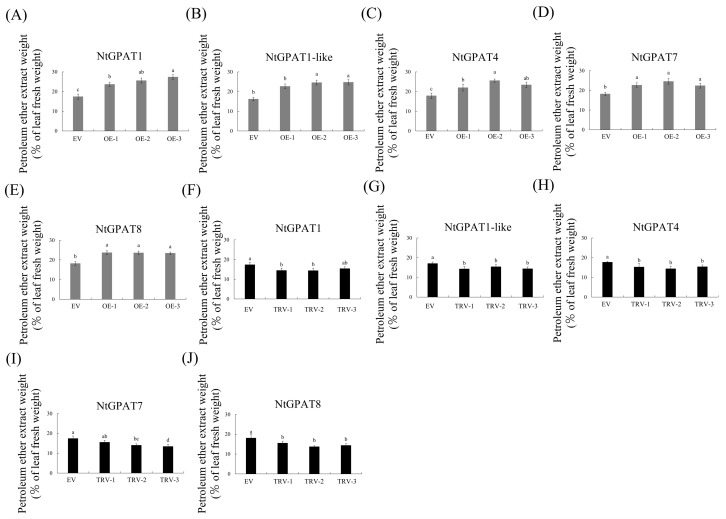
Transient silencing and overexpression of *NtGPATs* reduce and increase petroleum ether extract weight in tobacco, respectively. (**A**–**E**) Petroleum ether extract weight in leaves of *NtGPAT*-overexpressing tobacco plants. (**F**–**J**) Petroleum ether extract weight in leaves of transiently *NtGPAT*-silenced tobacco plants. EV: empty vector tobacco leaves (control). Different lowercase letters above bars indicate statistically significant differences among groups within the same developmental stage (Duncan’s multiple range test, *p* < 0.05).

To preliminarily assess the functional trends of the olive (*Olea europaea*) *OeGPAT* genes within a heterologous system, we employed *Nicotiana benthamiana* as a transient expression platform to modulate the activity of its endogenous homologs, *NtGPAT*. We constructed constitutive overexpression vectors (Figure 4A) and virus-induced gene silencing (VIGS) vectors (Figure 4B) for these genes. We subsequently quantified the relative expression levels of *NtGPAT1*, *NtGPAT1-like*, *NtGPAT4*, *NtGPAT5*, *NtGPAT6*, *NtGPAT7*, and *NtGPAT8* in tobacco leaves with stable overexpression (Figure 4C) and transient silencing (Figure 4D). RT-qPCR results showed that compared to the empty vector (EV), the expression levels of *GPATs* were significantly increased in the overexpression lines (Figure 4E–K), while they were markedly reduced in the transiently silenced plants (Figure 4L–R). These results demonstrate that the expression of *NtGPAT1*, *NtGPAT1-like*, *NtGPAT4*, *NtGPAT5*, *NtGPAT6*, *NtGPAT7*, and *NtGPAT8* was effectively upregulated or downregulated in the transformed tobacco plants, confirming their suitability for subsequent functional validation. Leaves were collected from tobacco plants overexpressing or transiently silencing *NtGPAT* genes, and the weight of petroleum ether extracts was analyzed. The results demonstrated that, compared with the EV control, the petroleum ether extract weight was significantly elevated in leaves of plants overexpressing *NtGPAT1*, *NtGPAT1-like*, *NtGPAT4*, *NtGPAT7*, and *NtGPAT8*, increasing by approximately 27.78–47.06% (Figure 5A–E). In contrast, the petroleum ether extract weight in leaves of transiently silenced tobacco plants was markedly reduced, decreasing by approximately 11.35–22.22% (Figure 5F–J). However, the overexpression or silencing of *NtGPAT5* and *NtGPAT6* genes in tobacco did not result in significant changes in petroleum ether extract weight (Supplementary Figure S1A–D). Based on the screening results obtained in *Nicotiana benthamiana*, their orthologous genes in olive, specifically *OeGPAT1.1*, *OeGPAT1.2*, *OeGPAT2*, *OeGPAT3*, *OeGPAT4*, *OeGPAT7*, and *OeGPAT8*, were designated as the core candidate genes for subsequent functional validation in olive.

### 2.4. Analysis of Expression Characteristics of Olive GPATs

**Figure 6:**
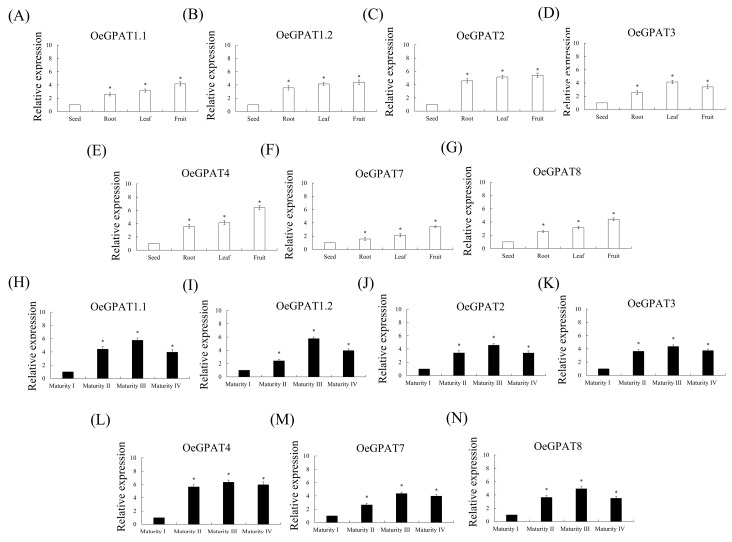
Analysis of the expression characteristics of *OeGPAT1.1*, *OeGPAT1.2*, *OeGPAT2*, *OeGPAT3*, *OeGPAT4*, *OeGPAT7*, and *OeGPAT8* genes in olive. (**A**–**G**) Analysis of the expression characteristics of *OeGPAT* genes in different tissues of olive. Error bars indicate ± SD with three replications. (**H**–**N**) Analysis of the expression characteristics of *OeGPAT* genes in olive fruits at different maturity levels. A single asterisk (*) specifies that the difference is significant between the treatment and the control at *p* < 0.05 (Duncan’s multiple range test).

We further investigated the expression patterns of *OeGPAT1.1*, *OeGPAT1.2*, *OeGPAT2*, *OeGPAT3*, *OeGPAT4*, *OeGPAT7*, and *OeGPAT8* in different tissues of Olive. The results indicated that most of these genes exhibited the highest expression levels in the fruit (Figure 6A–G). Additionally, the expression of these genes was significantly higher at Maturity stages II, III, and IV compared to Maturity I, with the highest expression observed at Maturity III (Figure 6H–N). In contrast, *OeGPAT5.1*, *OeGPAT5.2*, *OeGPAT5.3*, *OeGPAT6.1*, and *OeGPAT6.2* exhibited minimal variation across different tissues and developmental stages, showing no statistically significant changes (Supplementary Figure S2A–J).

### 2.5. Overexpression of OeGPATs in Arabidopsis Promotes Total Oil Accumulation

**Figure 7:**
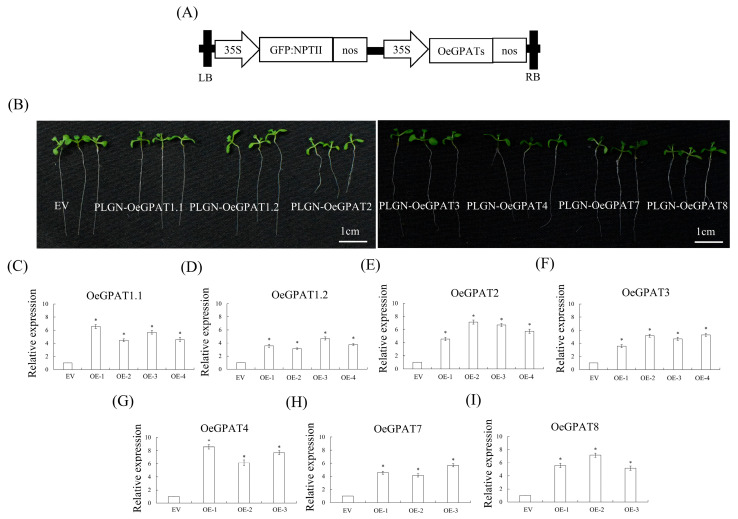
Heterologous overexpression and expression validation of *OeGPATs* in *Arabidopsis*. (**A**) Construction of the overexpression vector. (**B**) *Arabidopsis* plants overexpressing *OeGPATs*. (**C**–**I**) Relative gene expression levels of *OeGPATs* in the overexpression lines. EV: empty vector *Arabidopsis* (control). Scale bar: 1 cm. Error bars indicate ± SD with three replications. A single asterisk (*) specifies that the difference is significant between the treatment and the control at *p* < 0.05 (Duncan’s multiple range test).

**Figure 8:**
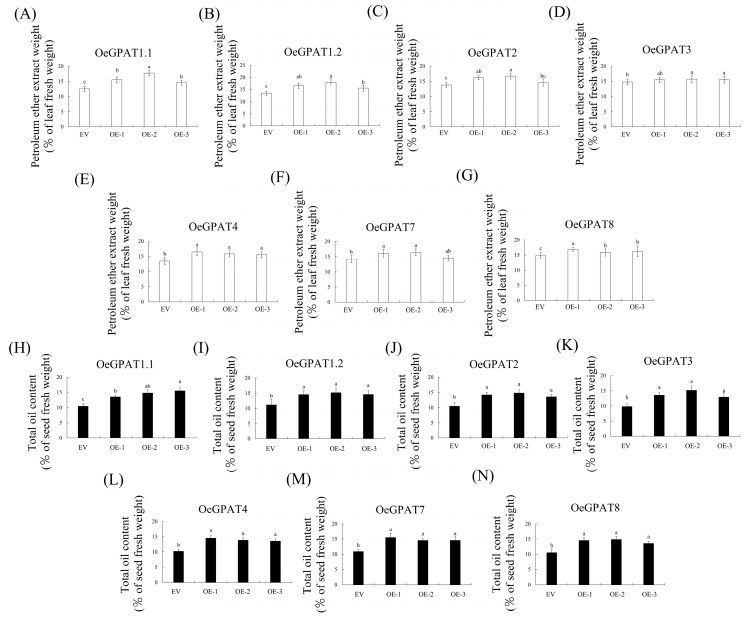
Overexpression of *OeGPATs* increases oil accumulation in *Arabidopsis*. (**A**–**G**) Petroleum ether extract weight in leaves of *OeGPAT*-overexpressing *Arabidopsis* lines. (**H**–**N**) Total oil content in seeds of *OeGPAT*-overexpressing *Arabidopsis* lines. EV: empty vector *Arabidopsis* (control). Different lowercase letters above bars indicate statistically significant differences among groups within the same developmental stage (Duncan’s multiple range test, *p* < 0.05).

Due to the lack of a stable genetic transformation system in olive, to further confirm the ability of *OeGPATs* to regulate oil accumulation, we constructed constitutive overexpression vectors for *OeGPAT1.1*, *OeGPAT1.2*, *OeGPAT2*, *OeGPAT3*, *OeGPAT4*, *OeGPAT7*, and *OeGPAT8* (Figure 7A), and heterologously overexpressed these genes in *Arabidopsis thaliana* (Figure 7B). RT-qPCR results showed that the expression of *OeGPAT1.1*, *OeGPAT1.2*, *OeGPAT2*, *OeGPAT3*, *OeGPAT4*, *OeGPAT7*, and *OeGPAT8* was significantly up-regulated in the transgenic *Arabidopsis* lines, confirming their suitability for subsequent functional validation (Figure 7C–I). Compared with the EV control, the petroleum ether extract weights in leaves (Figure 8A–G) and seeds (Figure 8H–N) of transgenic *Arabidopsis* overexpressing *OeGPAT1.1*, *OeGPAT1.2*, *OeGPAT2*, *OeGPAT3*, *OeGPAT4*, *OeGPAT7*, or *OeGPAT8* were all increased, showing significant enhancements of approximately 7.69–30.77% and 21.58–55.56%, respectively. Among them, as a high-oil tissue, changes in triglyceride content in seeds can be accurately assessed according to the ISO 659:1998 standard. In summary, the above results demonstrate that these genes positively regulate oil accumulation.

### 2.6. Transient Transformation of OeGPATs in Olive Affects Oil Accumulation

**Figure 9:**
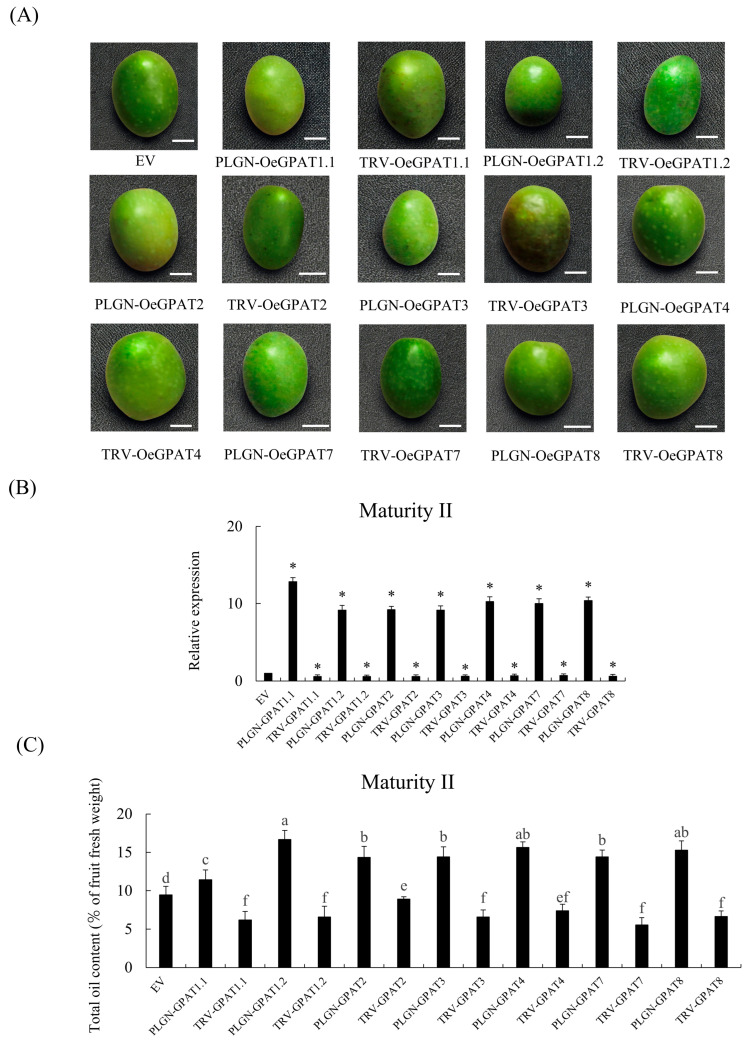
Quantitative analysis of total oils in olive fruits following transient transformation of *OeGPAT* genes at maturity stage II. (**A**) Phenotype of olive fruits at maturity stage II after transient transformation. Representative fruit bunches of EV, TRV-*OeGPATs*, and PLGN-*OeGPATs* fruits are shown. Scale bar: 1 cm. (**B**) Relative gene expression levels of *GPATs* in the overexpression and transient silencing olive fruits. (**C**) Quantitative analysis of total oil content in olive fruits subjected to transient overexpression or silencing of *OeGPATs* at maturity stage II. EV: empty vector olive fruits (control). Data are presented as mean ± SD (*n* ≥ 3). A single asterisk (*) specifies that the difference is significant between the treatment and the control at *p* < 0.05 (Duncan’s multiple range test). Different lowercase letters above bars indicate statistically significant differences among groups within the same developmental stage (Duncan’s multiple range test, *p* < 0.05).

**Figure 10:**
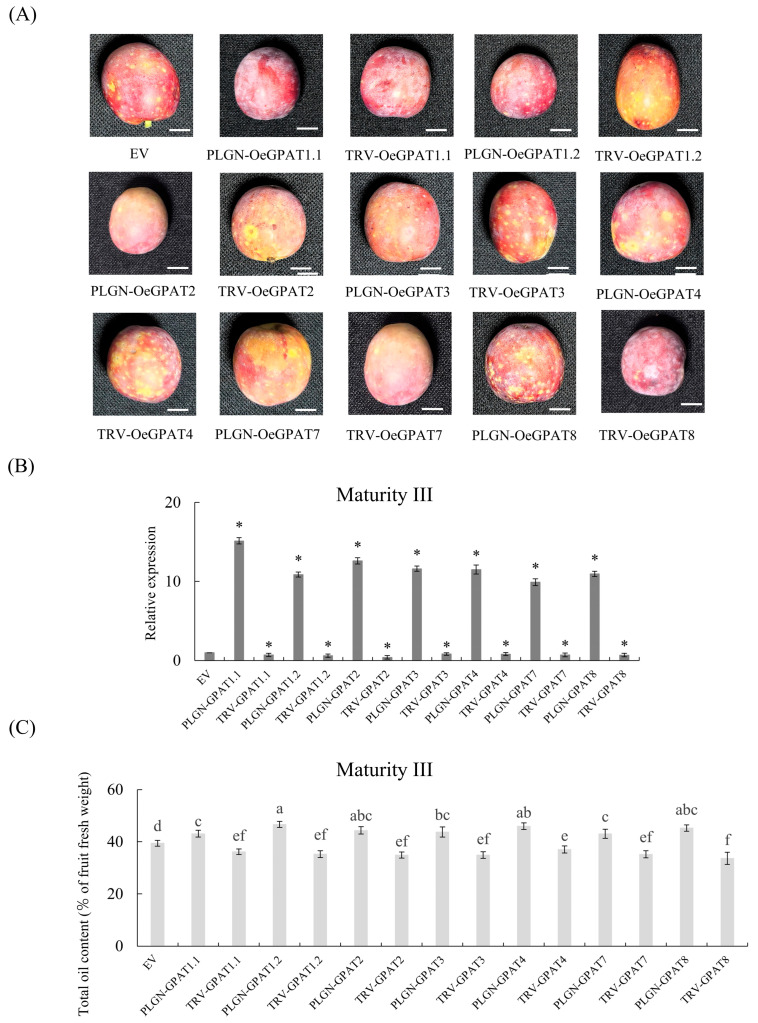
Quantitative analysis of total oils in olive fruits following transient transformation of *OeGPAT* genes at maturity stage III. (**A**) Phenotype of olive fruits at maturity stage III after transient transformation. Representative fruit bunches of EV, TRV-*OeGPATs*, and PLGN-*OeGPATs* fruits are shown. Scale bar: 1 cm. (**B**) Relative gene expression levels of *OeGPATs* in the overexpression and transient silencing olive fruits. (**C**) Quantitative analysis of total oil content in olive fruits subjected to transient overexpression or silencing of *OeGPATs* at maturity stage III. EV: empty vector olive fruits (control). Data are presented as mean ± SD (*n* ≥ 3). A single asterisk (*) specifies that the difference is significant between the treatment and the control at *p* < 0.05 (Duncan’s multiple range test). Different lowercase letters above bars indicate statistically significant differences among groups within the same developmental stage (Duncan’s multiple range test, *p* < 0.05).

**Figure 11:**
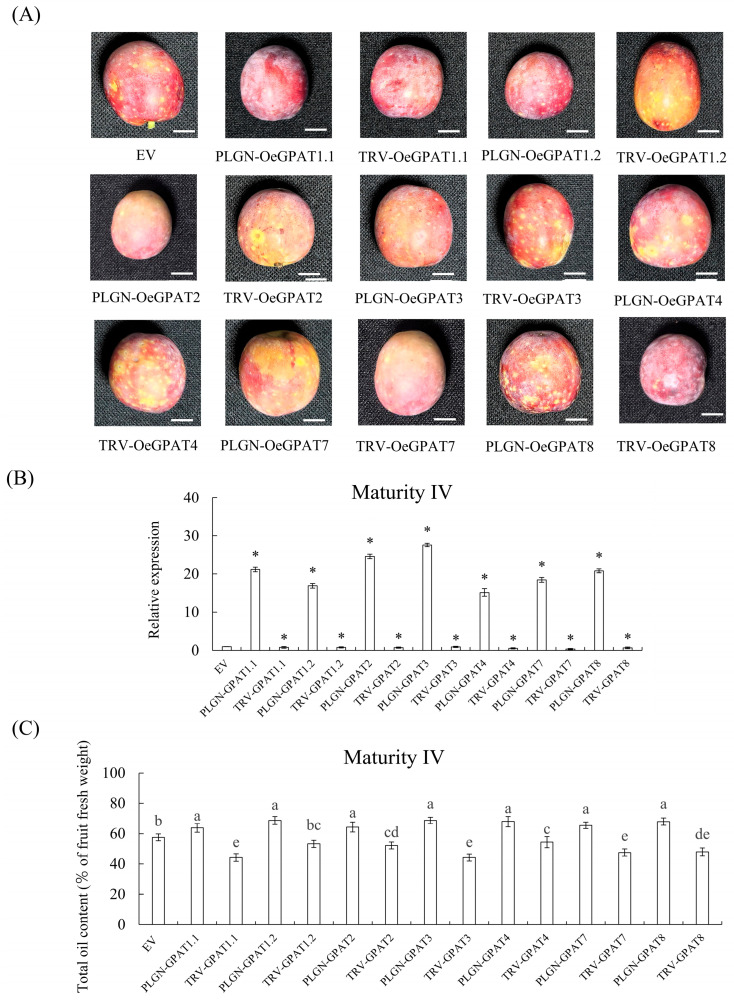
Quantitative analysis of total oils in olive fruits following transient transformation of *OeGPAT* genes at maturity stage IV. (**A**) Phenotype of olive fruits at maturity stage IV after transient transformation. Representative fruit bunches of EV, TRV-*OeGPATs*, and PLGN-*OeGPATs* fruits are shown. Scale bar: 1 cm. (**B**) Relative gene expression levels of *OeGPATs* in the overexpression and transient silencing olive fruits. (**C**) Quantitative analysis of total oil content in olive fruits subjected to transient overexpression or silencing of *OeGPATs* at maturity stage IV. EV: empty vector olive fruits (control). Data are presented as mean ± SD (*n* ≥ 3). A single asterisk (*) specifies that the difference is significant between the treatment and the control at *p* < 0.05 (Duncan’s multiple range test). Different lowercase letters above bars indicate statistically significant differences among groups within the same developmental stage (Duncan’s multiple range test, *p* < 0.05).

To validate the functions of these candidate genes within the native context of olive fruits, we conducted transient transformation assays in olive fruits at distinct developmental stages. Specifically, we introduced the pTRV2-*OeGPATs* silencing constructs and the PLGN-*OeGPATs* overexpression vectors into the fruits via injection (Figure 9, Figure 10 and Figure 11A). RT-qPCR analysis confirmed the successful manipulation of gene expression in transiently transformed olive fruits, with significant up-regulation of all seven *OeGPAT* genes under overexpression conditions and marked down-regulation upon gene silencing, thereby meeting the criteria for subsequent functional validation (Figure 9, Figure 10 and Figure 11B). Subsequent determination of oil content in the transformed fruits demonstrated that overexpression of *OeGPAT1.1*, *OeGPAT1.2*, *OeGPAT2*, *OeGPAT3*, *OeGPAT4*, *OeGPAT7*, and *OeGPAT8* elevated oil levels across maturity stages II, III, and IV of olive fruit. This corresponded to significant increases of approximately 27.78–77.78%, 7.59–14.68%, and 7.18–10.26%, respectively. Conversely, gene silencing reduced oil content at these same stages, resulting in significant decreases of approximately 24.44–38.89%, 5.06–10.13%, and 13.68–24.79%, respectively (Figure 9, Figure 10 and Figure 11C). We further examined the expression dynamics of *OeWRI1*-direct downstream target genes (*OeBCCP1*, *OeKASI*, *OeACP1*, *OeBCCP2*) in olive fruits across maturity stages II, III, and IV. RT-qPCR analysis revealed that these genes were significantly upregulated in transiently overexpressed fruits, whereas their expression was markedly reduced in transiently silenced fruits (Supplementary Figures S3–S5). These results are consistent with the findings in tobacco and *Arabidopsis*, confirming that *OeGPAT1.1*, *OeGPAT1.2*, *OeGPAT2*, *OeGPAT3*, *OeGPAT4*, *OeGPAT7*, and *OeGPAT8* positively regulate oil accumulation in olive fruit.

### 2.7. Overexpression of OeGPATs Alters the Fatty Acid Profile in Transgenic Arabidopsis Seeds

**Figure 12:**
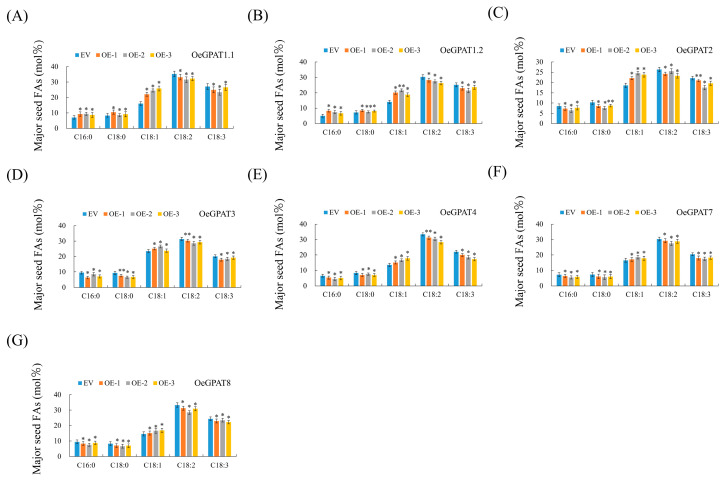
Distinct *OeGPAT* isoforms specifically modulate the fatty acid profile in transgenic *Arabidopsis* Seeds. (**A**–**G**) Relative abundance (mol%) of major fatty acid constituents in the EV and overexpression lines (OE-1, OE-2, and OE-3). A single asterisk (*) specifies that the difference is significant between the treatment and the control at *p* < 0.05; ** *p* ≤ 0.01. (Duncan’s multiple range test).

To elucidate the regulatory roles of *OeGPAT* genes in fatty acid composition, we performed quantitative profiling of fatty acids in seeds from EV controls and *OeGPAT*-overexpressing *Arabidopsis* lines. Compared with EV, lines overexpressing *OeGPAT1.1* and *OeGPAT1.2* exhibited a concurrent increase in the proportions of both monounsaturated fatty acid (C18:1) and saturated fatty acids (C16:0 and C18:0), whereas the proportion of polyunsaturated fatty acids (C18:2 and C18:3) decreased markedly (Figure 12A,B). In contrast, overexpression of *OeGPAT2*, *OeGPAT3*, *OeGPAT4*, *OeGPAT7*, or *OeGPAT8* resulted in a significant elevation of C18:1 levels, accompanied by pronounced reductions in both C18:2/C18:3 and saturated fatty acids (C16:0 and C18:0) (Figure 12C–G). These findings indicate that distinct *OeGPAT* isoforms likely possess divergent substrate specificities, which underpin the isoform-specific remodeling of fatty acid profiles in storage lipids.

## 3. Discussion

In a previous study, comprehensive widely targeted metabolomics and Illumina RNA-seq analyses were performed on five olive cultivars at different maturity stages to investigate the relationship between oil synthesis and the expression of related genes. The results showed that the total oil accumulation in the *Arbequina cultivar* at maturity stage III was significantly higher than that in other cultivars and at other maturity stages. Integrated transcriptome analysis further identified a oil biosynthesis pathway gene, *OeGPAT4*, whose expression level during different developmental stages of olive fruit exhibited a strong positive correlation with oil accumulation. This suggests that *OeGPAT* genes may function as key enzymatic genes regulating oil synthesis during olive fruit development and ripening. Therefore, based on the olive genome, this study identified and isolated members of the *OeGPAT* gene family, including *OeGPAT1.1*, *OeGPAT1.2*, *OeGPAT2*, *OeGPAT3*, *OeGPAT4*, *OeGPAT7*, and *OeGPAT8*, and further validated their biological functions in oil synthesis in olive fruit.

The first step in the biosynthesis of nearly all plant membrane phosphooils and storage triacylglycerols is catalyzed by GPAT, which acts as a pivotal rate-limiting enzyme. In plants, GPAT plays a crucial role in the biosynthesis of various glycerooils, including oil polymers, membrane-associated oils, and storage triacylglycerols. The *GPAT* gene family has been identified in multiple plant species, such as cotton (*Gossypium* spp.) [13], maize (*Zea mays*) [18], rice (*Oryza sativa*) [20], and *Arabidopsis* (*Arabidopsis thaliana*) [14]. However, no report has been published on the *GPAT* gene family in olive (*Olea europaea* L.), a distinctive energy oil crop. In this study, we performed comprehensive identification and analysis of the *GPAT* gene family in olive, with a particular focus on the functional characterization of key factors involved in oil biosynthesis.

A phylogenetic analysis of olive *GPATs* revealed a distinctive grouping pattern of GPAT proteins. While possessing conserved domains, different *GPAT* members also exhibited a degree of gene structure diversification, which may contribute to plant adaptation to diverse environments [29]. Members within the same *GPAT* gene cluster shared similar exon-intron structures, a pattern consistent with findings in other plant species, suggesting that diversification within the *GPAT* gene family may be associated with differences in gene architecture. Gene expression patterns can provide valuable clues for understanding gene function. For instance, in *Perilla frutescens*, the *PfGPAT9* gene shows higher expression levels in flowers and developing seeds [28]. In *peanut*, the *AhGPAT9* gene is most highly expressed in seeds, followed by leaves and shoots [30]. In *Euphorbia lathyris*, the *ElGPAT9* gene maintains high expression levels throughout various seed developmental stages [31]. In this study, the results showed that the seven *GPAT* genes were expressed to varying degrees across different olive tissues, with most exhibiting high expression in the fruit. Olive oil is extracted from the fruit, and a close relationship exists between fruit development and oil synthesis [8]. Maturity stage II occurs around 90 days after pollination, characterized by a gradual color change of the peel from green to red. Maturity stage III is reached approximately 110 days after flowering, when some fruits turn completely purple, indicating basic ripening. At maturity stage IV, the fruits are fully mature, with the peel turning black-purple, and oil accumulation peaks [9]. Using olive fruits at these three maturity stages as subjects, this study investigated the expression changes of the seven *OeGPAT* genes during different oil synthesis processes. The results indicated that all seven *OeGPATs* were expressed at varying levels in fruits across the three maturity stages, with most showing high expression at maturity stage III. These findings suggest that these seven *OeGPAT* genes play a significant regulatory role in oil synthesis and accumulation during olive fruit development.

In previous studies, knockout of the *AtGPAT9* gene led to a shriveled seed phenotype, indicating a significant reduction in seed oil content. Conversely, overexpression of the *AhGPAT9* gene in *Arabidopsis* resulted in increased accumulation of triacylglycerols in seeds, with the average linolenic acid content rising by approximately 14.91% [30]. Therefore, our study primarily focused on elucidating the role of *OeGPATs* in oil synthesis. Subsequently, we functionally characterized the *OeGPATs* using several approaches. In this study, we first employed a heterologous tobacco system for the high-throughput screening of candidate genes and found that the modulation of *NtGPAT1*, *NtGPAT1-like*, *NtGPAT4*, *NtGPAT7*, and *NtGPAT8* significantly affected petroleum ether extract weight (peak increases surpassed 47%). Subsequently, we conducted functional validation of the corresponding *OeGPAT* orthologs in both *Arabidopsis* and olive fruits. The results demonstrated that the overexpression of *OeGPAT1.1*, *OeGPAT1.2*, *OeGPAT2*, *OeGPAT3*, *OeGPAT4*, *OeGPAT7*, and *OeGPAT8* not only increased petroleum ether extract weight in *Arabidopsis* seeds and leaves (reached up to 55% above the control) but also significantly promoted oil accumulation during olive fruit ripening (increased by up to 77% relative to the control). This cross-species consistency indicates that these *GPAT* genes serve conserved and central functions in regulating oil biosynthesis. Notably, in this study, the gravimetric method based on petroleum ether extraction was employed to assess lipid changes in non-oil tissues (such as tobacco and *Arabidopsis* leaves). While this method accurately reflects triglyceride accumulation in high-oil tissues like olive fruits and *Arabidopsis* seeds, in leaf tissues, the primary components extracted are polar membrane lipids and co-extracted lipophilic metabolites (e.g., chlorophyll, sterols, cuticular waxes, etc.) rather than purely storage oils. Therefore, data from leaves should be interpreted as semi-quantitative indicators of overall changes in lipid metabolism. Nevertheless, the significant differences observed between transgenic lines and controls in this metric robustly demonstrate the positive regulatory role of *OeGPAT* genes in directing carbon flux toward lipid biosynthesis pathways. Future studies will employ thin-layer chromatography (TLC) or gas chromatography-mass spectrometry (GC-MS) to achieve precise quantification of specific lipid fractions.

Researchers have systematically elucidated key regulatory nodes within the lipid biosynthesis pathway. Oil synthesis and accumulation are governed by an intricate genetic regulatory network. The transcription factor WRINKLED1 (WRI1) functions as a “master switch” in this network, coordinately activating genes involved in fatty acid synthesis (FAS) and glycolysis to provide abundant precursors for triacylglycerol (TAG) assembly [16,32]. *WRI1* encodes an AP2 (APETALA2)-domain transcription factor that plays a pivotal role in oil regulation. Mutations in *WRI1* result in an approximately 80% reduction in seed oil content. Furthermore, *WRI1* activates the expression of several key genes encoding enzymes essential for oil biosynthesis, including *ACP1*, *BCCP*, *KASI*, and *PKP-β1*. Consequently, *WRI1* is recognized as a core transcription factor governing seed oil synthesis [33]. Its expression is activated by a suite of upstream transcription factors involved in seed development, such as *LEC1*, *LEC2*, *FUS3*, and *ABI3*, thereby promoting oil biosynthesis [34]. Transcriptomic analysis (RNA-seq) of seeds from varieties with varying oil contents across different developmental stages revealed that numerous oil synthesis-related genes (*ACP1*, *KASI*) are highly expressed during the early and middle stages of seed development. These findings unveil the fine-tuned regulatory network underlying flax seed oil accumulation [35]. In this study, the expression of *OeWRI1*-regulated target genes involved in oil biosynthesis (*OeBCCP1*, *OeKASI*, *OeACP1*, and *OeBCCP2*) responded dynamically to the transient expression of *GPATs* in olive fruits across three maturity stages. Transient overexpression of *OeGPATs* significantly upregulated the transcription of these genes, whereas transient silencing markedly reduced their expression levels. These findings indicate that the transient modulation of *OeGPATs* alters the transcriptional landscape of key fatty acid synthetic genes, thereby influencing total oil accumulation in olive fruits, although the precise molecular mechanisms underlying this regulatory cascade warrant further investigation.

In this study, transient transformation was performed on olive fruits at stages II, III, and IV, using fruits of the same developmental stage as controls to rule out interference from the endogenous developmental program on oil content quantification. Although endogenous ripening processes dominate the temporal dynamics of oil accumulation, alterations in *OeGPATs* expression within the same developmental window still significantly increased oil content, demonstrating that *OeGPATs* can autonomously regulate oil synthesis independently of the developmental background. As a rate-limiting enzyme initiating TAG synthesis, GPAT directly determines glycerol ester metabolic flux [14]. The phenotypic consistency observed in heterologous systems (tobacco, *Arabidopsis*) and *Nicotiana benthamiana* transient assays, coupled with the synchronous upregulation of fatty acid synthesis genes downstream of *OeWRI1*, confirms that the changes in oil content are attributable to *OeGPAT* enzymatic activity rather than secondary effects. Furthermore, the upregulation of *OeBCCP1*, *OeKASI*, *OeACP1*, and *OeBCCP2* by *OeGPAT* represents a direct cascade regulation within the lipid pathway, further amplifying its positive effect [35]. In summary, utilizing *OeGPATs* offers a promising strategy to further enhance oil content in olive fruit, thereby providing new insights for olive breeding and improvement.

GC-MS profiling revealed differential modulation of fatty acid composition by distinct *OeGPAT* isoforms in transgenic plants. Constitutive overexpression of *OeGPATs* generally led to a marked enrichment in oleic acid (C18:1), a phenotype consistent with the hypothesized role of GPAT as the rate-limiting enzyme in the Kennedy pathway. This effect is presumably mediated through the selective incorporation of specific acyl-CoA substrates, thereby dictating the initial assembly of the triacylglycerol (TAG) backbone. Given the premium quality of olive oil, which is characterized by its high oleic acid content, the observed substrate preference of *OeGPATs* positions them as ideal candidate genes for engineering improved oil quality in oilseed crops, specifically for elevating the proportion of monounsaturated fatty acids. Future investigations will include in vitro enzymatic assays to systematically determine the catalytic kinetic parameters of these isoforms toward acyl-CoAs varying in chain length and saturation degree.

While this study defines the role of seven *OeGPAT* genes in olive oil biosynthesis, three limitations remain. First, functional validation in olive relied on transient transformation, which yields no stable transgenic lines, precluding assessment of long-term effects across multiple growth cycles. Second, although synergistic expression between *OeGPATs* and the *OeWRI1* pathway was confirmed, their exact regulatory hierarchy and molecular interaction mechanisms, including whether *OeGPATs* are direct *OeWRI1* transcriptional targets or feedback-regulate *OeWRI1*, remain unresolved. Third, this study only addressed total oil content regulation, leaving unexplored the effects on fatty acid composition, oil quality, and abiotic stress tolerance, functions widely reported for plant *GPATs* [14]. Building on this, future work will: (1) establish stable olive transgenic lines to assess long-term breeding value; (2) dissect OeGPAT–OeWRI1 molecular mechanisms via Y1H, dual-LUC, and EMSA; and (3) evaluate impacts on fatty acid profiles, oil quality, and abiotic stress tolerance. In summary, *OeGPATs* are key positive regulators of oil accumulation, providing vital genetic resources for improving woody oil crops.

## 4. Materials and Methods

### 4.1. Plant Materials and Growth Conditions

This study focused on major olive cultivars, ‘Arbequina’ from an orchard in Jintang County, Sichuan Province. *Nicotiana benthamiana* was used for transient and stable genetic transformation, while *Arabidopsis thaliana* was employed for stable transformation. Transgenic plants were grown in a greenhouse under long-day conditions (16 h light/8 h dark) at 22 °C. For gene expression analysis, leaves from 4-week-old T3 generation *Arabidopsis* overexpression lines were collected. The experimental materials, including *N. benthamiana*, *A. thaliana*, and olive fruits, were provided by the Economic Forest Research Institute, Sichuan Academy of Forestry. With reference to the recommendations of the International Olive Council (IOC) and considering the ripening characteristics of the olive cultivar ‘Arbequina’, the fruit maturity stages are categorized as follows: Stage II is characterized by a yellow-green fruit skin; Stage III is defined by a partially purple or light-red fruit skin; and Stage IV is identified by a predominantly purple or deep-red fruit skin.

### 4.2. Identification and Physicochemical Characterization of OeGPAT Gene Family Members

Genomic sequences and annotation files for olive (*Olea europaea*), sesame (*Sesamum indicum*), and soybean (*Glycine max*) were retrieved from the NCBI database. The corresponding data for *Arabidopsis* thaliana were acquired from The *Arabidopsis* Information Resource (TAIR) [36]. To identify putative *OeGPAT* genes in olive, a hidden Markov model (HMM) profile search was conducted against the olive genome, using the known *Arabidopsis* GPAT protein sequences as a reference. Candidate genes identified through this screening were subsequently subjected to verification of the conserved *OeGPAT* domain using the Batch CD-Search tool (https://www.ncbi.nlm.nih.gov/Structure/cdd/wrpsb.cgi, accessed on 20 July 2026) [37]. The physicochemical properties of the confirmed olive GPAT proteins, including isoelectric point (pI), molecular weight (MW), grand average of hydropathicity (GRAVY), and instability index, were predicted using the ExPASy proteomics server (https://www.expasy.org/) [38].

### 4.3. Phylogenetic Analysis of the OeGPAT Gene Family

Protein sequences of *Arabidopsis thaliana* PAT family members, along with their conserved domain information, were retrieved from The *Arabidopsis* Information Resource (TAIR). Multiple sequence alignment of the conserved domains from both olive and *Arabidopsis* GPAT proteins was performed using the MUSCLE v5.1 software. Subsequently, a phylogenetic tree was constructed based on the aligned sequences using the Maximum Likelihood method implemented in the IQ-TREE 3.1.3 software [39], with branch support assessed by 1000 bootstrap replicates. The resulting tree was visualized and annotated using the Interactive Tree of Life (iTOL) online platform (v6, https://itol.embl.de/).

### 4.4. Chromosomal Localization, Gene Structure, and Promoter Cis-Acting Elements of OeGPAT Gene Family Members

Multiple sequence alignment of conserved domain sequences of olive GPAT family proteins was performed using DNAMAN 10.3 and Jalview 2.11 software. Gene structure was predicted and analyzed via the GSDS database (http://gsds.gao-lab.org/). Gene structure information was extracted from the genome annotation file and visualized using TBtools-II software [40]. Conserved motifs and domains were analyzed using the online tool MEME and the InterPro database (https://www.ebi.ac.uk/interpro/, accessed on 20 July 2026) [41], with the number of motifs set to 10. Visualization was conducted using the gene structure module in TBtools-II. A 2000 bp sequence upstream of the start codon for each olive *GPAT* family gene was extracted from the olive genome. These promoter sequences were submitted to the PlantCARE website (http://bioinformatics.psb.ugent.be/webtools/plantcare/html/, accessed on 20 July 2026) for prediction of cis-acting elements. The results were subsequently visualized using TBtools-II software.

### 4.5. Chromosomal Localization and Collinearity Analysis of OeGPAT Gene Family Members

Chromosomal localization information of the *OeGPAT* gene family members and the lengths of their respective chromosomes were obtained by screening the olive genome annotation file. The MG2C online tool (http://mg2c.iask.in/mg2c_v2.1/index.html, accessed on 20 July 2026) was used for visualization, and relevant parameters were adjusted to optimize the layout, resulting in a standardized chromosome distribution map. Collinearity analysis was performed using the MCScanX tool in TBtools software to identify collinear gene pairs and tandem duplicated gene pairs. The results were visualized using TBtools.

### 4.6. Gene Cloning and Recombinant Vector Construction

Specific primers (Supplementary Table S1) were designed for 7 *NtGPAT* and *OeGPAT* genes based on their coding sequence (CDS) regions, using cDNA derived from both tobacco leaves and olive fruits as templates. PCR amplification was performed using 2× TransStart^®^ FastPfu Fly PCR SuperMix (AS231, TransGen Biotech, Beijing, China). The amplified CDS fragments of these genes were each ligated into the constitutive plant expression vector PLGN. The recombinant plasmids were then transformed into *Escherichia coli* DH5α competent cells. Positive clones were identified by PCR and sequencing, and the confirmed recombinant plasmids were subsequently introduced into *Agrobacterium tumefaciens* GV3101 for genetic transformation of *Arabidopsis* and tobacco.

Based on the online SGN VIGS Tool (https://vigs.solgenomics.net/), the optimal gene silencing sequences for tobacco leaves and olive fruits *GPATs* were selected. Upstream and downstream primers were then designed using Primer Premier 5 software, with the primer sequences provided in Supplementary Table S1. The TRV2 vector plasmid was first linearized by digestion with *Eco*RI and *Bam*HI restriction enzymes. Subsequently, the target gene fragments were ligated into the linearized vector using a one-step cloning method. Finally, the ligation product was transformed into *E. coli* DH5α competent cells. Following plating and culture, single colonies were selected and inoculated in liquid broth for amplification.

### 4.7. Stable Transformation of Nicotiana benthamiana and Arabidopsis thaliana

The recombinant plasmids were introduced into sterile *N. benthamiana* leaf discs via *Agrobacterium*-mediated transformation. Transformed shoots were subsequently selected on medium containing hygromycin B (5 mg L^−1^) or phosphinothricin (PPT, 1 mg L^−1^). For *Arabidopsis*, the binary vector was introduced into EV plants (Col-0) using the floral-dip method [42,43]. T_0_ seeds were screened for positive transformants under blue light based on green fluorescent protein fluorescence, and positive lines were confirmed by PCR genotyping. Transgenic plants were verified at both the genomic and transcriptional levels by PCR. Finally, rooted positive homozygous tobacco plantlets and confirmed *Arabidopsis* plants were transferred to a nursery substrate for further cultivation and subsequent total oil content analysis.

### 4.8. Transient Transformation of Nicotiana benthamiana and Olive Fruits

The TRV2 vector, TRV2-*GPATs* constructs, and the TRV1 vector were individually introduced into *Agrobacterium tumefaciens* strain EHA105. The transformed agrobacteria harboring the expression vectors were cultured on LB medium supplemented with 50 μg/mL kanamycin, 50 μg/mL rifampicin, and 50 μg/mL streptomycin. Cultures were grown at 28 °C with shaking at 200 rpm for approximately 16 h until the OD_600_ reached 2.0. Bacterial cells were collected by centrifugation at 5000 rpm for 10 min. The supernatant was discarded, and the pellet was resuspended in freshly prepared MMA buffer (10 mM MgCl_2_, 10 mM MES/KOH pH 5.6, 150 μM acetosyringone) and diluted to an OD_600_ of 0.4.

Tobacco seedlings with four true leaves were selected. A mixed *Agrobacterium* suspension (TRV2 + TRV1, or TRV2-*GPATs* + TRV1) was infiltrated into the abaxial side of the leaves using a sterile syringe until all four leaves were fully infiltrated. After 12 h of dark incubation, the plants were lightly sprayed with water to aid recovery and then transferred to normal growth conditions. Leaves were collected 3 days after treatment, immediately flash-frozen in liquid nitrogen, and stored at −80 °C for subsequent experiments.

For the VIGS experiment in olive fruits, the method described for sweet cherry [44], Loquat [45], citrus [46], and pear [47] was followed. At each maturity stage (II, III, and IV), multiple randomly selected fruits were injected with 20 μL of the *Agrobacterium* mixture (TRV2 + TRV1, or TRV2-*GPATs* + TRV1) near the equatorial region using an INJEX-300 injector (INJEX, Berlin, Germany). Immediately after injection, the fruits were bagged to maintain humidity. Tissue from the injection site was collected on the 5th day, flash-frozen in liquid nitrogen, and stored at −80 °C for subsequent analysis.

The procedure for the transient overexpression experiment in olive fruits was identical to that of the VIGS experiment.

### 4.9. DNA, RNA Extraction and Quantitative Real-Time PCR (qRT-PCR) Validation

Total RNA was extracted using the EASYspin Plus Plant RNA Kit (Aidlab, Beijing, China), and first-strand cDNA was synthesized with the PrimeScript™ RT Reagent Kit (TaKaRa, Beijing, China). Genomic DNA from olive fruits was extracted following the instructions of the M5 CTAB Plant Genomic DNA Extraction Kit (Mei5 Biotechnology Co., Ltd., Beijing, China). Total RNA was isolated from olive fruit tissues using TRIzol reagent (Tiangen, Beijing, China), following the manufacturer’s protocol. First-strand cDNA was synthesized from the extracted RNA using the RevertAid First Strand cDNA Synthesis Kit (Thermo Fisher Scientific, Waltham, MA, USA). qRT-PCR reactions were carried out in triplicate for each biological sample (three independent biological replicates per condition) using SYBR Green Master Mix (Tiangen, Beijing, China) on a Bio-Rad CFX 96 Real-Time PCR System (Bio-Rad, Hercules, CA, USA). The sequences of the gene-specific primers used are provided in Supplementary Table S1. The GAPDH gene was used as an internal control for normalization. Relative gene expression levels were calculated using the comparative 2^−ΔΔCT^ method. Data are expressed as the mean ± standard deviation (SD), and the results were visualized using bar graphs created in Microsoft Excel.

### 4.10. Determination of Total Oil Content

Oil content was determined according to the standard method ISO 659:1998 [48]. All samples were freshly harvested tissues (olive fruits, tobacco leaves, *Arabidopsis* leaves, and seeds), which were immediately flash-frozen in liquid nitrogen and stored long-term at −80 °C. Prior to extraction, samples were ground into a homogeneous powder under liquid nitrogen. Approximately 2 g of each sample was placed in a Soxhlet extractor and extracted with petroleum ether for 6 h. The extracts were concentrated by rotary evaporation at 40 °C under 25 Torr to remove the solvent, followed by drying under a stream of nitrogen gas to obtain the oil fraction, which was stored at −20 °C until further analysis [49]. All oil content data were calculated based on fresh weight (FW). Each experiment included at least three independent biological replicates (*n* ≥ 3), and each biological sample was analyzed in triplicate (technical replicates) to ensure reproducibility and reliability.

### 4.11. Measurement of Fatty Acids (FAs)

The extraction and analysis of fatty acids were performed following the method described by Mu et al. [50]. For the preparation of fatty acid methyl esters (FAMEs), approximately 10 mg of mature seeds were incubated in 1 M HCl/methanol at 80 °C for 2.5 h. Subsequently, 1 mL of *n*-hexane and 2 mL of 0.9% (*w*/*v*) NaCl solution were added, and the mixture was vortexed vigorously for 60 s to ensure thorough mixing. Following centrifugation at 600× *g* for 5 min, the upper organic phase was collected. Methyl heptadecanoate was employed as the internal standard for quantitative analysis by gas chromatography (GC). Chromatographic analysis was conducted on a Shimadzu GC-2014 system (Shimadzu, Kyoto, Japan) equipped with a flame ionization detector (FID) and a Supelcowax-10 polar capillary column (Sigma-Aldrich, Taufkirchen, Germany). The oven temperature program was as follows: initial temperature held at 160 °C for 1 min, increased to 240 °C at a rate of 4 °C min^−1^, and finally maintained at 240 °C for 16 min. Individual fatty acid components were identified based on their retention times, and the corresponding peak areas were integrated and quantified using GC Solution 2.4 software (Shimadzu, Kyoto, Japan). Ultimately, the concentrations of all fatty acid species were normalized to the internal standard.

### 4.12. Statistical Analysis

All statistical analyses were performed using SPSS software (version 26.0, IBM Corp., Armonk, NY, USA) and visualized with Microsoft Excel 2019. Data are presented as the mean ± standard deviation (SD) based on at least three independent biological replicates (*n* ≥ 3), with each biological sample analyzed in triplicate (technical replicates) to ensure reproducibility. Significant differences among groups within the same developmental stage or treatment were assessed by one-way analysis of variance (ANOVA) followed by Duncan’s multiple range test at a significance level of *p* < 0.05. Asterisks (*) in figures denote statistically significant differences between the treatment and the control.

## 5. Conclusions

Through systematic identification and functional analysis of the olive *GPAT* gene family, this study successfully cloned seven key genes, including *OeGPAT1.1*, *OeGPAT1.2*, *OeGPAT2*, *OeGPAT3*, *OeGPAT4*, *OeGPAT7*, and *OeGPAT8*. The results showed that these genes are highly expressed in olive fruit, with their transcript levels significantly upregulated at fruit maturity stages II, III, and IV. Heterologous overexpression of these seven genes notably increased oil content in the leaves and seeds of *Arabidopsis*. Transient transformation in olive fruit further confirmed that they positively regulate oil biosynthesis by activating *OeBCCP1*, *OeKASI*, *OeACP1*, and *OeBCCP2*. This study reveals the molecular mechanism by which the olive *GPAT* gene family regulates oil accumulation and provides important candidate genes and a theoretical foundation for the metabolic engineering improvement of oil yield and quality in oil crops.

## Data Availability

The datasets generated during and/or analyzed during the current study are available from the corresponding author on reasonable request.

## Supplementary Materials

The following supporting information can be downloaded at: https://www.mdpi.com/article/10.3390/plants15152295/s1, Supplementary Table S1: The primers used in the study; Supplementary Table S2: Sequence similarity of Olea europaea GPAT with GPAT homologs from Arabidopsis thaliana and Nicotiana tabacum; Supplementary Table S3: Protein Sequences and Annotations of Olea europaea GPAT Gene Family Members; Supplementary Table S4: Best Homologous Gene Matches of Olea europaea GPAT Genes in Other Species; Supplementary Table S5: Transcript and Protein Sequence Information of GPAT Gene Family in Olea europaea, Nicotiana tabacum, and Arabidopsis thaliana; Supplementary Table S6: Distribution and Classification of Cis-acting Elements in the Promoters of GPAT Gene Family; Supplementary Table S7: Amino Acid Sequences of Conserved Motifs in the GPAT Protein Family; Supplementary Figure S1: The transient silencing and overexpression of *NtGPAT5* and *NtGPAT6* have no significant effect on petroleum ether extract weight in tobacco; Supplementary Figure S2: Analysis of the expression characteristics of *OeGPAT5.1*, *OeGPAT5.2*, *OeGPAT5.3*, *OeGPAT6.1*, and *OeGPAT6.2* genes in olive; Supplementary Figure S3: Relative expression levels of *OeBCCP1*, *OeKASI*, *OeACP1* and *OeBCCP2* genes in olive fruits subjected to transient transformation of *OeGPATs*; Supplementary Figure S4: Expression analysis of key oil synthesis genes in olive fruits subjected to transient transformation of *OeGPATs*; Supplementary Figure S5: Expression response of oil synthesis-related genes in olive fruits at maturity stage IV.

## References

[1] Diez C.M., Trujillo I., Martinez-Urdiroz N., Barranco D., Rallo L., Marfil P., Gaut B.S. (2015). Olive domestication and diversification in the Mediterranean Basin. New Phytol..

[2] Cruz F., Julca I., Gómez-Garrido J., Loska D., Marcet-Houben M., Cano E., Galán B., Frias L., Ribeca P., Derdak S. (2016). Genome sequence of the olive tree, *Olea europaea*. GigaScience.

[3] Owen R.W., Giacosa A., Hull W.E., Haubner R., Würtele G., Spiegelhalder B., Bartsch H. (2000). Olive-oil consumption and health: The possible role of antioxidants. Lancet Oncol..

[4] Rizzo M., Godino G., Perri E., Zelasco S., Lombardo L. (2024). Development of a rapid and fruit-saving method for fatty acid composition analysis in olive: A comparative study on 27 cultivars. Front. Plant Sci..

[5] Estruch R., Ros E., Salas-Salvadó J., Covas M.-I., Corella D., Arós F., Gómez-Gracia E., Ruiz-Gutiérrez V., Fiol M., Lapetra J. (2018). Primary Prevention of Cardiovascular Disease with a Mediterranean Diet Supplemented with Extra-Virgin Olive Oil or Nuts. N. Engl. J. Med..

[6] Hernández M.L., Sicardo M.D., Alfonso M., Martínez-Rivas J.M. (2019). Transcriptional regulation of stearoyl-acyl carrier protein desaturase genes in response to abiotic stresses leads to changes in the unsaturated fatty acids composition of olive mesocarp. Front. Plant Sci..

[7] Ye Z., Yu J., Yan W., Zhang J., Yang D., Yao G., Liu Z., Wu Y., Hou X. (2021). Integrative iTRAQ-based proteomic and transcriptomic analysis reveals the accumulation patterns of key metabolites associated with oil quality during seed ripening of Camellia oleifera. Hortic. Res..

[8] Conde C., Delrot S., Gerós H. (2008). Physiological, biochemical and molecular changes occurring during olive development and ripening. J. Plant Physiol..

[9] Migliorini M., Cherubini C., Mugelli M., Gianni G., Trapani S., Zanoni B. (2011). Relationship between the oil and sugar content in olive oil fruits from Moraiolo and Leccino cultivars during ripening. Sci. Hortic..

[10] Wang X., Liang H., Guo D., Guo L., Duan X., Jia Q., Hou X. (2019). Integrated analysis of transcriptomic and proteomic data from tree peony (*P. ostii*) seeds reveals key developmental stages and candidate genes related to oil biosynthesis and fatty acid metabolism. Hortic. Res..

[11] Mak H.Y., Ouyang Q., Tumanov S., Xu J., Rong P., Dong F., Lam S.M., Wang X., Lukmantara I., Du X. (2021). AGPAT2 interaction with CDP-diacylglycerol synthases promotes the flux of fatty acids through the CDP-diacylglycerol pathway. Nat. Commun..

[12] Graham I.A. (2008). Seed storage oil mobilization. Annu. Rev. Plant Biol..

[13] Cui Y., Ma J., Liu G., Wang N., Pei W., Wu M., Li X., Zhang J., Yu J. (2019). Genome-Wide Identification, Sequence Variation, and Expression of the Glycerol-3-Phosphate Acyltransferase (GPAT) Gene Family in Gossypium. Front. Genet..

[14] Shockey J., Regmi A., Cotton K., Adhikari N., Browse J., Bates P.D. (2016). Identification of Arabidopsis GPAT9 (At5g60620) as an Essential Gene Involved in Triacylglycerol Biosynthesis. Plant Physiol..

[15] He Z., Zhang P., Jia H., Zhang S., Nishawy E., Sun X., Dai M. (2024). Regulatory mechanisms and breeding strategies for crop drought resistance. New Crops.

[16] Liu F., Xi M., Liu T., Wu X., Ju L., Wang D. (2024). The central role of transcription factors in bridging biotic and abiotic stress responses for plants’ resilience. New Crops.

[17] Chen X., Snyder C.L., Truksa M., Shah S., Weselake R.J. (2011). sn-Glycerol-3-phosphate acyltransferases in plants. Plant Signal. Behav..

[18] Zhu T., Wu S., Zhang D., Li Z., Xie K., An X., Ma B., Hou Q., Dong Z., Tian Y. (2019). Genome-wide analysis of maize GPAT gene family and cytological characterization and breeding application of ZmMs33/ZmGPAT6 gene. Theor. Appl. Genet..

[19] Men X., Shi J., Liang W., Zhang Q., Lian G., Quan S., Zhu L., Luo Z., Chen M., Zhang D. (2017). Glycerol-3-Phosphate Acyltransferase 3 (OsGPAT3) is required for another development and male fertility in rice. J. Exp. Bot..

[20] Waschburger E., Kulcheski F.R., Veto N.M., Margis R., Margis-Pinheiro M., Turchetto-Zolet A.C. (2018). Genome-wide analysis of the Glycerol-3-Phosphate Acyltransferase (GPAT) gene family reveals the evolution and diversification of plant GPATs. Genet. Mol. Biol..

[21] Zheng Z., Xia Q., Dauk M., Shen W., Selvaraj G., Zou J. (2003). Arabidopsis AtGPAT1, a member of the membrane-bound glycerol-3-phosphate acyltransferase gene family, is essential for tapetum differentiation and male fertility. Plant Cell.

[22] Singer S.D., Chen G., Mietkiewska E., Tomasi P., Jayawardhane K., Dyer J.M., Weselake R.J. (2016). Arabidopsis GPAT9 contributes to synthesis of intracellular glycerooils but not surface oils. J. Exp. Bot..

[23] Beisson F., Li Y., Bonaventure G., Pollard M., Ohlrogge J.B. (2007). The acyltransferase GPAT5 is required for the synthesis of suberin in seed coat and root of *Arabidopsis*. Plant Cell.

[24] Li X.-C., Zhu J., Yang J., Zhang G.-R., Xing W.-F., Zhang S., Yang Z.-N. (2012). Glycerol-3-phosphate acyltransferase 6 (GPAT6) is important for tapetum development in Arabidopsis and plays multiple roles in plant fertility. Mol. Plant.

[25] Yang W., Simpson J.P., Li-Beisson Y., Beisson F., Pollard M., Ohlrogge J.B. (2012). A Land-plant-specific glycerol-3-phosphate acyltransferase family in Arabidopsis: Substrate specificity, sn-2 preference, and evolution. Plant Physiol..

[26] Jayawardhane K.N., Singer S.D., Weselake R.J., Chen G. (2018). Plant sn-Glycerol-3-Phosphate Acyltransferases: Biocatalysts Involved in the Biosynthesis of Intracellular and Extracellular Oils. Lipids.

[27] Lv Y., Zhang X., Luo L., Yang H., Li P., Zhang K., Liu F., Wan Y. (2020). Characterization of glycerol-3-phosphate acyltransferase 9 (AhGPAT9) genes, their allelic polymorphism and association with oil content in peanut (*Arachis hypogaea* L.). Sci. Rep..

[28] Zhou Y., Huang X., Hu T., Chen S., Wang Y., Shi X., Yin M., Li R., Wang J., Jia X. (2023). Genome-Wide Analysis of Glycerol-3-Phosphate Acyltransferase (GPAT) Family in *Perilla frutescens* and Functional Characterization of PfGPAT9 Crucial for Biosynthesis of Storage Oils Rich in High-Value Oils. Int. J. Mol. Sci..

[29] Safder I., Shao G., Sheng Z., Hu P., Tang S. (2021). Identification and Analysis of the Structure, Expression and Nucleotide Polymorphism of the GPAT Gene Family in Rice. Plant Gene.

[30] Shen Y., Shen Y., Liu Y., Bai Y., Liang M., Zhang X., Chen Z. (2023). Characterization and Functional Analysis of AhGPAT9 Gene Involved in Oil Synthesis in Peanut (*Arachis hypogaea* L.). Front. Plant Sci..

[31] Wang Z., Su Y., Wang L., Ma L., Sun Y., Li R., Ge L. (2024). Characterization of GPAT Gene Family in *Euphorbia lathyris* L. and Elucidating the Role of ElGPAT9 in the Biosynthesis of Oils and Pollen Viability. Ind. Crop. Prod..

[32] Khan M., Zhang J., Zhang Y., Zhang Y., Tang C., Wang J. (2026). Metabolic engineering of vegetative organs for enhanced oil accumulation in biofuel feedstocks. Crop. J..

[33] Wei W., Wang L., Tao J., Zhang W., Chen S., Song Q., Zhang J. (2025). The comprehensive regulatory network in seed oil biosynthesis. J. Integr. Plant Biol..

[34] Yang Y., Kong Q., Lim A.R., Lu S., Zhao H., Guo L., Yuan L., Ma W. (2022). Transcriptional regulation of oil biosynthesis in seed plants: Current understanding, applications, and perspectives. Plant Commun..

[35] Yadav H.K., Singh N., Singh B., Kaur V., Sawant S.V. (2025). Telomere-to-telomere genome assembly of linseed (*Linum usitatissimum* L.) for functional genomics and accelerated genetic improvement. Plant Biotechnol. J..

[36] Rhee S.Y., Beavis W., Berardini T.Z., Chen G., Dixon D., Doyle A., Garcia-Hernandez M., Huala E., Lander G., Montoya M. (2003). The Arabidopsis Information Resource (TAIR): A model organism database providing a centralized, curated gateway to Arabidopsis biology, research materials and community. Nucleic Acids Res..

[37] Marchler-Bauer A., Anderson J.B., Chitsaz F., Derbyshire M.K., DeWeese-Scott C., Fong J.H., Geer L.Y., Geer R.C., Gonzales N.R., Gwadz M. (2009). CDD: Specific functional annotation with the Conserved Domain Database. Nucleic Acids Res..

[38] Duvaud S., Gabella C., Lisacek F., Stockinger H., Ioannidis V., Durinx C. (2021). Expasy, the Swiss Bioinformatics Resource Portal, as designed by its users. Nucleic Acids Res..

[39] Nguyen L.-T., Schmidt H.A., Von Haeseler A., Minh B.Q. (2015). IQ-TREE: A fast and effective stochastic algorithm for estimating maximum-likelihood phylogenies. Mol. Biol. Evol..

[40] Chen C.J., Chen H., Zhang Y., Thomas H.R., Frank M.H., He Y.H., Xia R. (2020). TBtools: An Integrative Toolkit Developed for Interactive Analyses of Big Biological Data. Mol. Plant.

[41] Mulder N.J. (2005). InterPro, progress and status in 2005. Nucleic Acids Res..

[42] Clough S.J., Bent A.F. (1998). Floral dip: A simplified method for Agrobacterium-mediated transformation of *Arabidopsis thaliana*. Plant J..

[43] Zhang X., Henriques R., Lin S.-S., Niu Q.-W., Chua N.-H. (2006). Agrobacterium-mediated transformation of Arabidopsis thaliana using the floral dip method. Nat. Protoc..

[44] Qi X., Liu C., Song L., Li Y., Li M. (2017). PaCYP78A9, a Cytochrome P450, Regulates Fruit Size in Sweet Cherry (*Prunus avium* L.). Front. Plant Sci..

[45] Chi Z., Zhao M., Wang L., Hu Q., Wang S., Guo Q., Jing D., Liang G., Xia Y. (2025). EjNAC25, a NAC transcription factor in early-maturing seedless triploid loquat, promotes sugar and malic acid accumulation by activating EjNI and EjtDT2. Postharvest Biol. Technol..

[46] Gong J., Tian Z., Qu X., Meng Q., Guan Y., Liu P., Chen C., Deng X., Guo W., Cheng Y. (2021). Illuminating the cells: Transient transformation of citrus to study gene functions and organelle activities related to fruit quality. Hortic. Res..

[47] Zhou F., Wang X., Wang Y., Wang Z., Zhang H., Zhang C., Zhai R., Yang C., Wang Z., Xu L. (2026). The PbNAC56-PbMYB31 regulatory module mediates volatile ester biosynthesis in pear. Hortic. Plant J..

[48] (1998). Oilseeds—Determination of Hexane Extract (or Light Petroleum Extract), Called “Oil Content”.

[49] Matthäus B., Musazcan Özcan M. (2015). Oil content, fatty acid composition and distributions of vitamin-E-active compounds of some fruit seed oils. Antioxidants.

[50] Mu J., Tan H., Zheng Q., Fu F., Liang Y., Zhang J., Yang X., Wang T., Chong K., Wang X.-J. (2008). LEAFY COTYLEDON1 is a key regulator of fatty acid biosynthesis in Arabidopsis. Plant Physiol..

