# Supplementary material for: Identification and Functional Characterization of the GPAT Gene Family in Regulating Oil Biosynthesis in Olive
Source: Plants (Basel). 2026 Jul 27;15(15):2295. doi: 10.3390/plants15152295 (PMC13468065; doi:10.3390/plants15152295)
Supplement: Supplementary file 1 [file plants-15-02295-s001.zip › Supplementary figures.pdf]

Identification and Functional Characterization of the GPAT Gene Family in Regulating Oil Biosynthesis in Olive

Shengjia Huang, Lihua Wang, Min Ye, Wanbo Wu, Hui Luo, Lingfan Yu, Pijun Li, Yang Huang and Jincheng Du *

Sichuan Academy of Forestry, Chengdu 610081, China; huangshengjia0206@163.com (S.H.); wwlhbb@163.com (L.W.); yemincome@163.com (M.Y.); 66880027@163.com (W.W.); paul779375110@163.com (H.L.); yulingfan328@163.com (L.Y.); barophiles@139.com (P.L.); huangyang0927@163.com (Y.H.)

* Correspondence: 18190770069@163.com

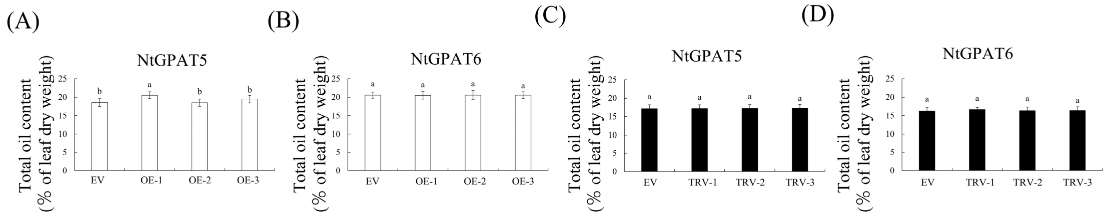

Supplementary Figure S1. The transient silencing and overexpression of *NtGPAT5* and *NtGPAT6* have no significant effect on petroleum ether extract weight in tobacco. (A–B) Petroleum ether extract weight in leaves of *NtGPAT5* and *NtGPAT6*-overexpressing tobacco plants. **(C–D)** Petroleum ether extract weight content in leaves of transiently *NtGPAT5* and *NtGPAT6*-silenced tobacco plants. EV: empty vector tobacco leaves (control). Different lowercase letters above bars indicate statistically significant differences among groups within the same developmental stage (Duncan's multiple range test, $p < 0.05$).

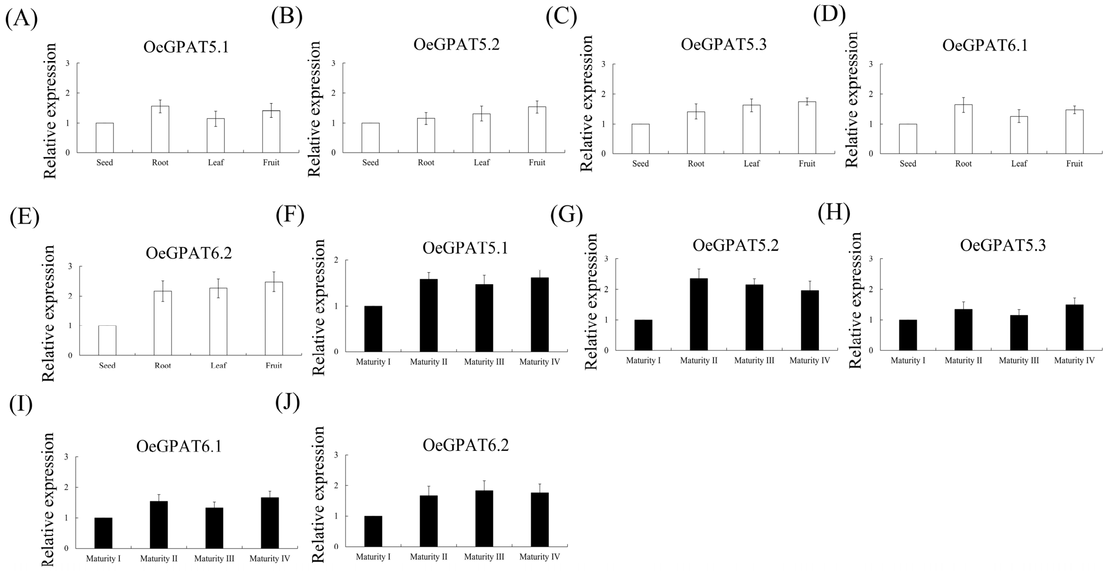

Supplementary Figure S2. Analysis of the expression characteristics of *OeGPAT5.1*, *OeGPAT5.2*, *OeGPAT5.3*, *OeGPAT6.1*, and *OeGPAT6.2* genes in olive. (A) Analysis of the expression characteristics of *OeGPATs* genes in different tissues of olive. Error bars indicate ± SD with three replications. **(B)** Analysis of the expression characteristics of *OeGPATs* genes in olive fruits at different maturity levels. A single asterisk (*) specifies that the difference is significant between the treatment and the control at $p < 0.05$ (Duncan's multiple range test).

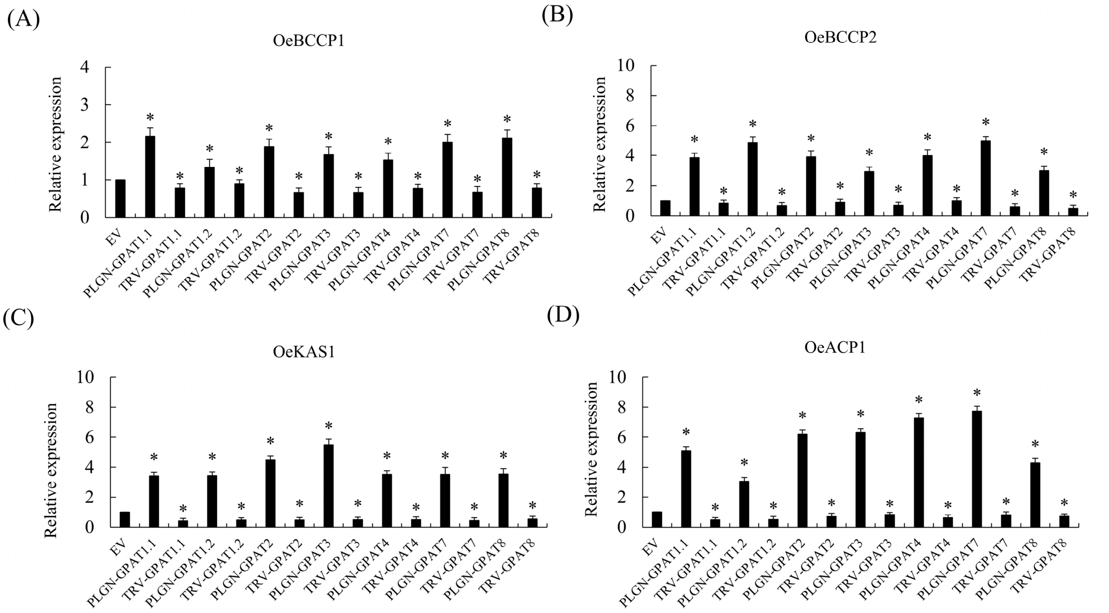

Supplementary Figure S3. Relative expression levels of *OeBCCP1, OeKASI, OeACP1 and OeBCCP2* genes in olive fruits subjected to transient transformation of *OeGPATs*. RT-qPCR analysis showed that at maturity stage II, the expression levels of key oil biosynthesis genes, *OeBCCP1* **(A)**, *OeKASI* **(B)**, *OeACP1* **(C)**, and *OeBCCP2* **(D)**, were significantly up-regulated in fruits treated with transient overexpression of *OeGPATs* mediated by the PLGN vector, compared with EV. EV: empty vector olive fruits (control). In contrast, their expression was significantly down-regulated in fruits subjected to transient silencing of *OeGPATs* mediated by the TRV vector. A single asterisk (*) specifies that the difference is significant between the treatment and the control at $p < 0.05$ (Duncan's multiple range test).

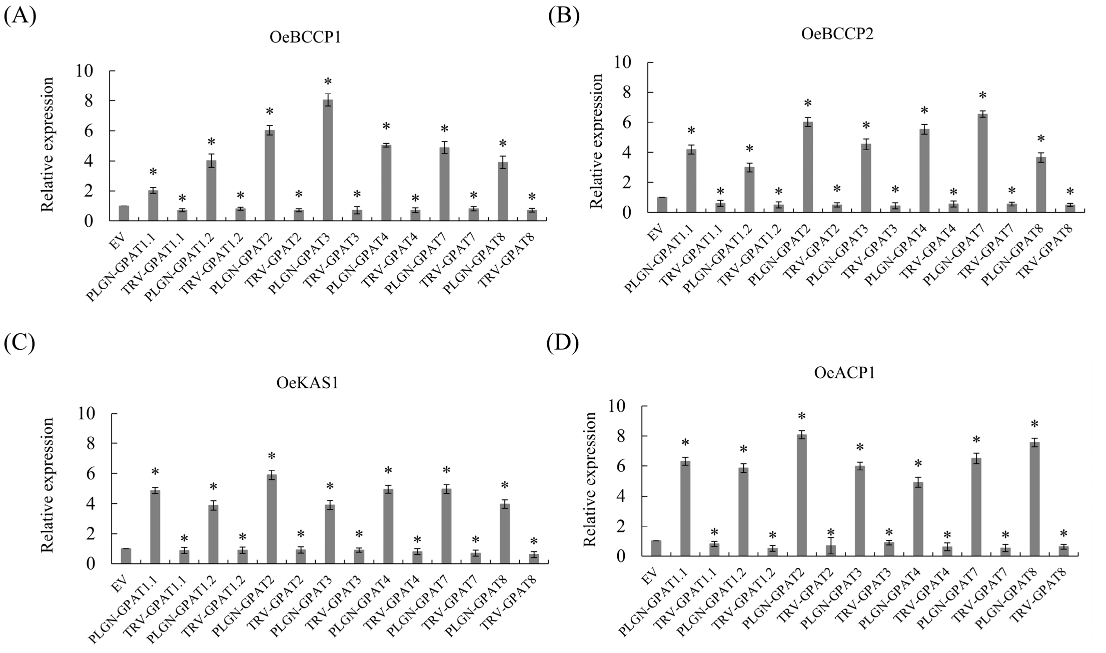

Supplementary Figure S4. Expression analysis of key oil synthesis genes in olive fruits subjected to transient transformation of *OeGPATs*. RT-qPCR analysis revealed that at maturity stage III, transient overexpression of *OeGPATs* mediated by the PLGN vector significantly up-regulated the expression levels of key oil biosynthesis genes, *OeBCCP1* **(A)**, *OeKASI* **(B)**, *OeACP1* **(C)**, and *OeBCCP2* **(D)**. In contrast, transient silencing of *OeGPATs* mediated by the TRV vector markedly suppressed the expression of these genes. EV: empty vector olive fruits (control). A single asterisk (*) specifies that the difference is significant between the treatment and the control at $p < 0.05$ (Duncan's multiple range test).

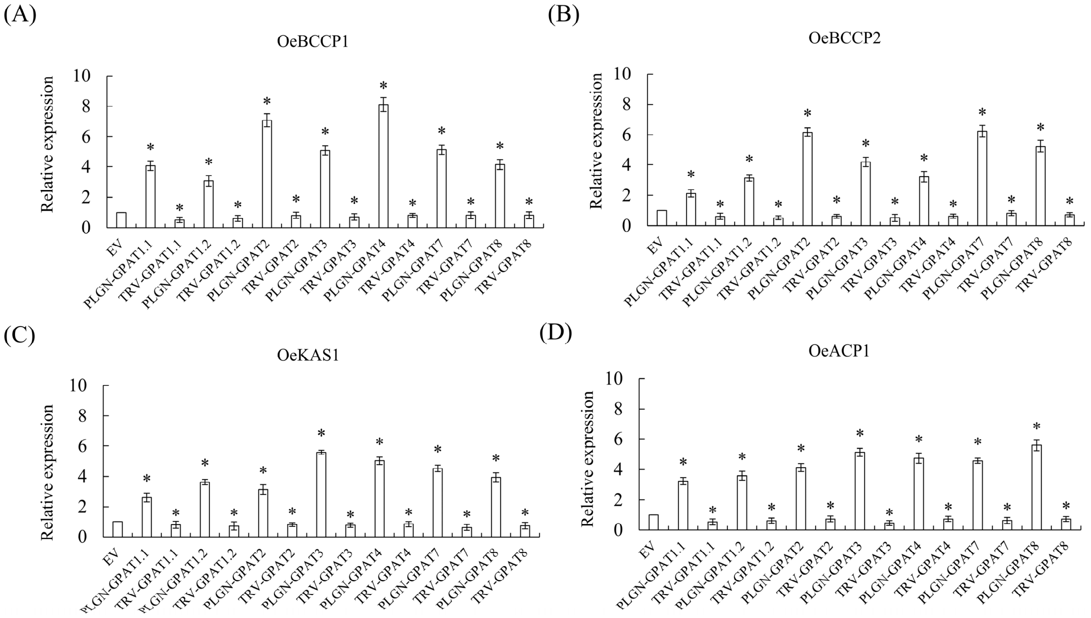

Supplementary Figure S5. Expression response of oil synthesis-related genes in olive fruits at maturity stage IV. RT-qPCR analysis showed that at maturity stage IV, transient overexpression of *OeGPATs* (mediated by the PLGN vector) significantly up-regulated the transcript levels of key oil synthesis genes, *OeBCCP1* **(A)**, *OeKASI* **(B)**, *OeACP1* **(C)**, and *OeBCCP2* **(D)**. In contrast, transient silencing of *OeGPATs* (mediated by the TRV vector) markedly suppressed the expression of these genes. EV: empty vector olive fruits (control). A single asterisk (*) specifies that the difference is significant between the treatment and the control at $p < 0.05$ (Duncan's multiple range test).
